# 

**Published:** 1842-10-08

**Authors:** 


**CONTENTS OF No. 41.**

Dr. Wallace's note on an Antidote to Corrosive Sublimate, - - 641
FOREIGN CRRESPONDENCE : Letter from Paris, - . - - - 642
EDITORIAL : Dr. Clymer's correction, - - - . - - 645
Ligature of the Primitive Iliac Artery, - - - - 645
ANALECTA : Dr. Ward on the Treatment of Asphygia by Flagellation, 647
Dr. Nottingham's New Splint, - - - - - - 648
M. Payan on the Employment of the Ergot of Rye in cases of Paraplegia, - - - - - - - - - 650
Iodine for the Dropsy, after Scarlet Fever, - - - 651
The employment of Strychnia in Amaurosis, - - - 651
The Independence of the Fœtal Circulation, - - - 651
Disease of the Nasal Fossæ, - - - - - - 652
Conversion of Nerves into Fat, - - - - - - 653
M. Liebig on the Preparation of Cyanide of Potassium, - 653
Peritonitis from Perforation of the Appendix Vermiformis, 655

